# Critical Role of Regulator G-Protein Signaling 10 (RGS10) in Modulating Macrophage M1/M2 Activation

**DOI:** 10.1371/journal.pone.0081785

**Published:** 2013-11-21

**Authors:** Jae-Kyung Lee, Jaegwon Chung, George T. Kannarkat, Malú G. Tansey

**Affiliations:** Department of Physiology, Emory University School of Medicine, Atlanta, Georgia, United States of America; Temple University School of Medicine, United States of America

## Abstract

Regulator of G protein signaling 10 (RGS10), a GTPase accelerating protein (GAP) for G alpha subunits, is a negative regulator of NF-κB in microglia. Here, we investigated the role of RGS10 in macrophages, a closely related myeloid-derived cell type. Features of classical versus alternative activation were assessed *in Rgs10-/-* peritoneal and bone marrow-derived macrophages upon LPS or IL-4 treatments, respectively. Our results showed that *Rgs10-/-* macrophages produced higher levels of pro-inflammatory cytokines including TNF, IL-1β and IL-12p70 in response to LPS treatment and exerted higher cytotoxicity on dopaminergic MN9D neuroblastoma cells. We also found that *Rgs10-/-* macrophages displayed a blunted M2 phenotype upon IL-4 priming. Specifically, *Rgs10-/-* macrophages displayed lower YM1 and Fizz1 mRNA levels as measured by QPCR compared to wild type macrophages upon IL-4 treatment and this response was not attributable to differences in IL-4 receptor expression. Importantly, phagocytic activities of *Rgs10-/-* macrophages were blunted in response to IL-4 priming and/or LPS treatments. However, there was no difference in chemotaxis between *Rgs10-/-* and WT macrophages. Our data indicate that *Rgs10-/-* macrophages displayed dysregulated M1 responses along with blunted M2 alternative activation responses, suggesting that RGS10 plays an important role in determining macrophage activation responses.

## Introduction

Macrophages respond to their microenvironment through polarizing into either a classical or alternatively activated phenotype [1]. Classically activated (M1) macrophages are critical effector cells involved in killing microorganisms. M1 activation is induced by bacterial lipopolysaccharide (LPS), interferon-γ (IFN-γ), or tumor necrosis factor (TNF) and is associated with the production of large amounts of pro-inflammatory cytokines, including TNF and IL-6 [2]. This activation is mediated by several signaling pathways including nuclear factor kappa-light-chain-enhancer of activated B cells (NF-κB), and mitogen-activated protein kinase (MAPK). In contrast, the resolution phase of inflammation is driven by alternatively activated (M2) macrophages. M2 macrophages are involved in restoring tissue homeostasis after injury by switching to a gene profile that supports repair and tissue reconstruction. The switch to an alternative phenotype is induced by multiple factors from Th2-polarized T lymphocytes recruited to the injury site in which IL-4 and IL-13 are the predominant induction signals [2]. Treatment of murine macrophages with IL-4 or IL-13 induces up-regulation of arginase [3], YM1 (chitinase-like lectin), and FIZZ1 (resistin-like secreted protein) expression [4,5]. IL-4 leads to dimerization of IL-4 receptor and activates the JAK family of tyrosine kinases, leading to phosphorylation of the receptor cytoplasmic tails and exposure of docking sites for STATs, primarily STAT6 [6]. 

The regulator of G-protein signaling (RGS) family of proteins are GTPase accelerating proteins (GAPs) that negatively regulate G-protein coupled receptor (GPCR) signaling by increasing the rate of GTP hydrolysis by Gα [7,8]. More than thirty genes make up the mammalian RGS protein family and they can be divided into 9 subfamilies based on sequence homology within the RGS domain or on structural similarities. RGS10 belongs to the R12 subfamily and is expressed in brain, thymus, and lymph nodes [9,10]. In humans, susceptibility genes for age-related maculopathy (ARM), a photoreceptor degenerative disease associated with microgliosis, map to the same region of human chromosome 10q26 as the RGS10 gene [28,29], suggesting that loss of RGS10 may predispose an organism to neurodegenerative disease. Also, a novel polymorphism in the RGS domain RGS10 was reported in a Japanese patient with schizophrenia [30], but the functional significance of RGS10 and its protein expression levels in psychiatric disorders is still unknown. In addition, RGS10 and RGS17 transcript levels have been reported to be significantly lower in ovarian cancer cells that are resistant to chemotherapy compared to chemo-sensitive cells [31]. The role of RGS10 in regulating macrophage activation and the deleterious downstream effect of dysregulated immune responses on neuronal homeostasis may in part underlie these associations in humans.

Phosphorylation of RGS10 by PKA at Ser-168 induces translocation of RGS10 from the plasma membrane and the cytosol into the nucleus [11] but it is not known whether it participates in regulation of gene transcription. Previous work from our group implicated RGS10 in immune cell function in the central nervous system (CNS). Specifically, we reported that *Rgs10-/-* mice displayed increased microglial burden in the CNS and that induction of chronic systemic inflammation in these mice resulted in degeneration of dopamine-producing neurons in the substantia nigra of the midbrain, a histopathological hallmark of parkinsonism [12]. Given the role of RGS10 in modulating NF-kB signaling and the pro-inflammatory phenotype of *Rgs10-/-* microglia [13], herein we investigated whether RGS10 modulates macrophage activation and polarization as an additional potential explanation for the neurodegenerative phenotype induced by chronic peripheral LPS injections. Our results support a role for RGS10 as an important determinant of macrophage activation during inflammatory responses and suggest that RGS10 deficiency in the CNS as well as in the peripheral immune system may contribute to increased vulnerability to inflammation-induced degeneration in the CNS. 

## Materials and Methods

### Animals

Generation of *Rgs10-/-* mice has been described previously [13]. Four to six month old male mice were used for experiments. Age- and gender- matched wild-type (WT) mice were used as controls. Experimental procedures involving use of animal tissue were performed in accordance with the NIH Guidelines for Animal Care and Use and approved by the Institutional Animal Care and Use Committee at Emory University School of Medicine in Atlanta, GA. 

### Tissue Culture

 Mouse peritonitis was induced by i.p. injection of 4 % thioglycolate broth (Sigma-Aldrich) in mice. Peritoneal macrophages were isolated 3 days after i.p. injection. Macrophages were plated in 6 well-plates at a density of 1 × 10^6^ cells per well in DMEM medium supplemented with 10% FBS. Bone-marrow-derived macrophages (BMDMs) were prepared from WT and *Rgs10-/-* mice by maturing bone marrow cells in DMEM containing 10% FBS and 20% of L929 conditioned media for 14 days [14]. BMDMs were cultured in medium without L-929 medium for 2 days prior to experiments to deprive them of macrophage colony stimulating factor (M-CSF). 

### Multiplexed ELISAs

 Macrophage cells were either treated with LPS (100 ng/mL) for 24 hours or primed for 48 hours with IL-4, IL-13 or IFN-γ (10 ng/mL) and then challenged with LPS (100 ng/mL) for 24 hours. Supernatants were harvested, centrifuged at 8000g, and stored at -80 °C to measure the production of cytokines and chemokines (mouse IFN-γ, IL-1β, IL-6, IL-10, IL-12, KC, and TNF) using a multiplexed immunoassay per the manufacturer's instructions (Meso-Scale Discovery, Gaithersburg, MD).

### Quantitative Real-time RT-PCR (QPCR)

 Macrophages were primed for 48 hours with IL-4, IL-13 or IFN-γ (10 ng/mL) and challenged with LPS (100 ng/mL) for 24 hours. Total RNA was isolated from cells in culture using the RNeasy isolation kit (Qiagen), treated with DNaseI, and reverse transcribed using Superscript II RNase H- reverse transcriptase (Invitrogen). QPCR was performed using SYBR Green in a 384-well format using an ABI Prism 7900HT Fast Detection System (Applied Biosystems, Foster City). Oligonucleotide primers for QPCR were obtained from Integrated DNA Technologies (Coralville). Primers used in the study were validated by analysis of template titration and dissociation curves [15]. mRNA expression levels of YM and Fizz1 were normalized to those of the geometric mean of mouse house-keeping genes actin, cyclophilin B and GAPDH. Data are representative of at least two independent experiments. Values represent the mean value of triplicate samples +/- SEM. mRNA levels of RGS family were presented as threshold of Cycles (Ct) values. 

### Target effector assays

Assays were performed as described previously [12] using MN9D neuroblastoma cells (target) and macrophages (effector cells). Macrophage cells (1 × 10^5^) were treated with LPS (100 ng/mL) for 24 hours and conditioned medium was collected. Conditioned medium was transferred to MN9D cells plated in flat-bottomed 96-well plates at a density of 7 × 10^3^ cells/well for 48 hours. CellTiter 96 Aqueous Assay (Promega) was used to measure metabolic activity of MN9D cells during the last 2-4 hours of a two-day culture as a measure of cell viability. 

### Nitric Oxide (NO) Assays

Peritoneal macrophage cells (1 × 10^5^) were treated for 48 hours with IL-4, IL-13 or IFN-γ (10 ng/mL) priming and then challenged with LPS (100 ng/mL) for 18 hours. The culture supernatant (100 µL) was added to 100 µL of Griess reagent (Promega) and the absorbance at 540 nm was measured on a microplate spectrophotometer reader. 

### Phagocytosis Assays

Macrophages were plated at a density of 1 × 10^5^ cells per well in 96-well plates and allowed to adhere for 8 hours. Upon adherence, cells were primed with PBS or IL-4 (10 ng/mL) for 48 hours followed by various treatments with LPS (10 ng/mL or 1 µg/mL) for 18 hours. Phagocytic activity measured using the Vibrant Phagocytosis Assay using fluorescently labeled *E. coli* particles (Invitrogen). 

### Chemotaxis Assay

Macrophages were fluorescently labeled using 200 nM of Mitotracker Red CM_2_-XROS (Molecular Probes, Invitrogen) for 30 min at 37 °C. Cells were washed in serum-free media and then plated at 2 x 10^5^ cells per well in the tissue culture insert provided in the HTS FluoroBlok Multiwell Insert System (BD Falcon). The inserts were placed in a feeder tray with 1 mL of serum-free media in each well. Cells were allowed to adhere for 3 hours in the incubator and then inserts were transferred to a seeder plate which contained LPS (1 µg/mL), medium containing 10 % FBS or LPS (1 µg/mL) plus medium containing 20 % FBS for 18 hours. Plate was read using a bottom-reading fluorescent plate reader at 585 nm excitation/ 620 nm emission wavelenghts. Each experimental condition was performed in quadruplicate. 

### Western blot analysis

Macrophages were plated at the density of 500,000 cells per well in 6-well plate. Cells were lysed with 300 µL of 2x Laemmli sample buffer containing 1 % NP-40, 10 mM Tris pH 7.4, 150 mM NaCl, 100 μg/mL PMSF, and protease inhibitor mix (Sigma). Lysates were loaded on precast SDS-PAGE gels (Bio-Rad), transferred onto PDVF membranes (Millipore), and probed with anti-STAT6, anti-phospho STAT6, YM1, Arg 1, anti-p65, anti-p50 and α-tubulin antibodies (Santa Cruz Biotechnology) plus the appropriate HRP-conjugated secondary antibody (1:5000, Jackson ImmunoResearch Lab, West Grove, PA). Immunoreactive bands were visualized on a Syngene geldocumentation box with SuperSignal West Femto HRP substrate (Thermo Fisher Scientific, Rockford, IL) according to the manufacturer’s instructions. Membranes were stripped with 0.2 M glycine, 1 % SDS and 0.1 % Tween-20, pH 2.2 and re-probed as necessary. 

### Flow Cytometry

For surface staining, cells were washed with FACS buffer (1mM EDTA, 0.01% sodium azide, 0.1% BSA, 0.02 M phosphate, 0.15 M NaCl, pH 7.2) and then stained for 20 minutes with fluorophore-conjugated antibodies: anti-CD11b-PE (ebiosciences), anti-CD14-PerCP-Cy5.5 (ebiosciences), anti-CD45-APC (ebiosciences), anti-IL-4-receptor-PE (ebiosciences). If applicable, intracellular staining was then performed using Invitrogen Fixation and Permeabilization Media with goat anti-RGS10 primary antibody (Santa Cruz Biotechnology) and donkey anti-goat Fc FITC-conjugated secondary antibody (Santa Cruz Biotechnology). After staining, cells were washed and then fixed with 1% paraformaldehyde for 30 minutes. After washing, cells were stored in FACS buffer until analysis on a FACS Calibur (BD Biosciences). Data analysis was performed on FlowJo software.

### Statistical Analysis

Effect of genotype and treatment were analyzed by two-way ANOVA followed by the Bonferroni *post hoc* test for *p* values significance. Differences among groups were analyzed using one-way ANOVA. One way ANOVA followed by Tukey’s post-hoc test, Values expressed are the group mean +/- SEM; *p < 0.05; **p< 0.01;***p <0.001 compared within the group. ^#^ *p* < 0.05; ^##^ *p*< 0.01; ^###^ *p* <0.001 compared between the groups.

## Results

### RGS10 expression and regulation in mouse macrophages

**Figure 1:**
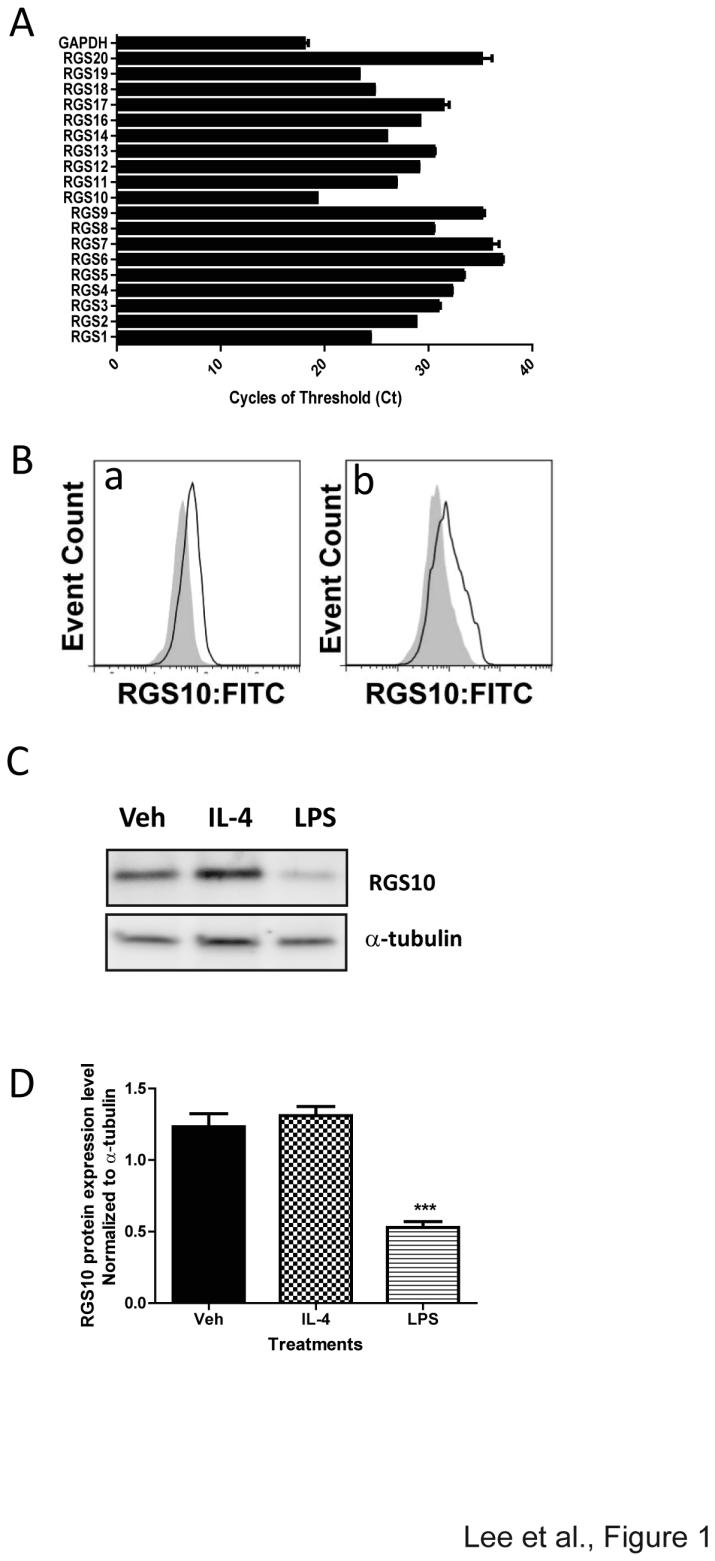
Macrophages express RGS10 and its expression is regulated by LPS. A) RGS mRNA levels in mouse BMDMs were assessed by quantitative PCR. Cycle threshold (Ct) values reveal that RGS10 is the most abundantly expressed gene. B) Peripheral blood monocytes and splenic macrophages express RGS10 at levels detectable by intracellular flow cytometry staining. Intracellular RGS10 expression in CD11b+CD14+CD45+ monocytes (a) and splenic macrophages (b) was measured by flow cytometry in Rgs10-/- (shaded) and WT (unshaded) mice. C) Representative western blot data and D) quantitative analysis of expression of RGS10 protein levels in bone marrow-derived macrophages (BMDM) upon LPS (100 ng/mL) treatment. The results represent the mean +/- SEM of three independent experiments. Student t-test; **, *p*< 0.01 or ***, *p* <0.001.

Quantitative real-time PCR analysis revealed that RGS10 is the most abundant RGS family proteins in macrophages (Figure 1A) and flow cytometry analysis (Figure 1B) confirmed RGS10 protein expression in both peripheral blood monocytes and splenic macrophages. Next, we measured RGS10 protein expression level in bone marrow-derived macrophages (BMDM) using western blot analysis (Figure 1C, D). To test whether RGS10 protein expression is regulated by M1 or M2 polarization, we used LPS stimulation as an M1 stimulus and IL-4 as an M2 stimulus on bone-marrow derived macrophages from wild type (WT) mice. Expression of RGS10 was significantly attenuated following LPS treatment, but it was not changed by IL-4 treatment (Figure 1C, D). Time course experiments demonstrated a significant decrease in RGS10 expression by 48 hours after stimulation. Similar observations were obtained in peritoneal macrophages (data not shown). 

### *Rgs10-/-* macrophages display increased pro-inflammatory cytokine production upon lipopolysaccharide treatment

**Figure 2:**
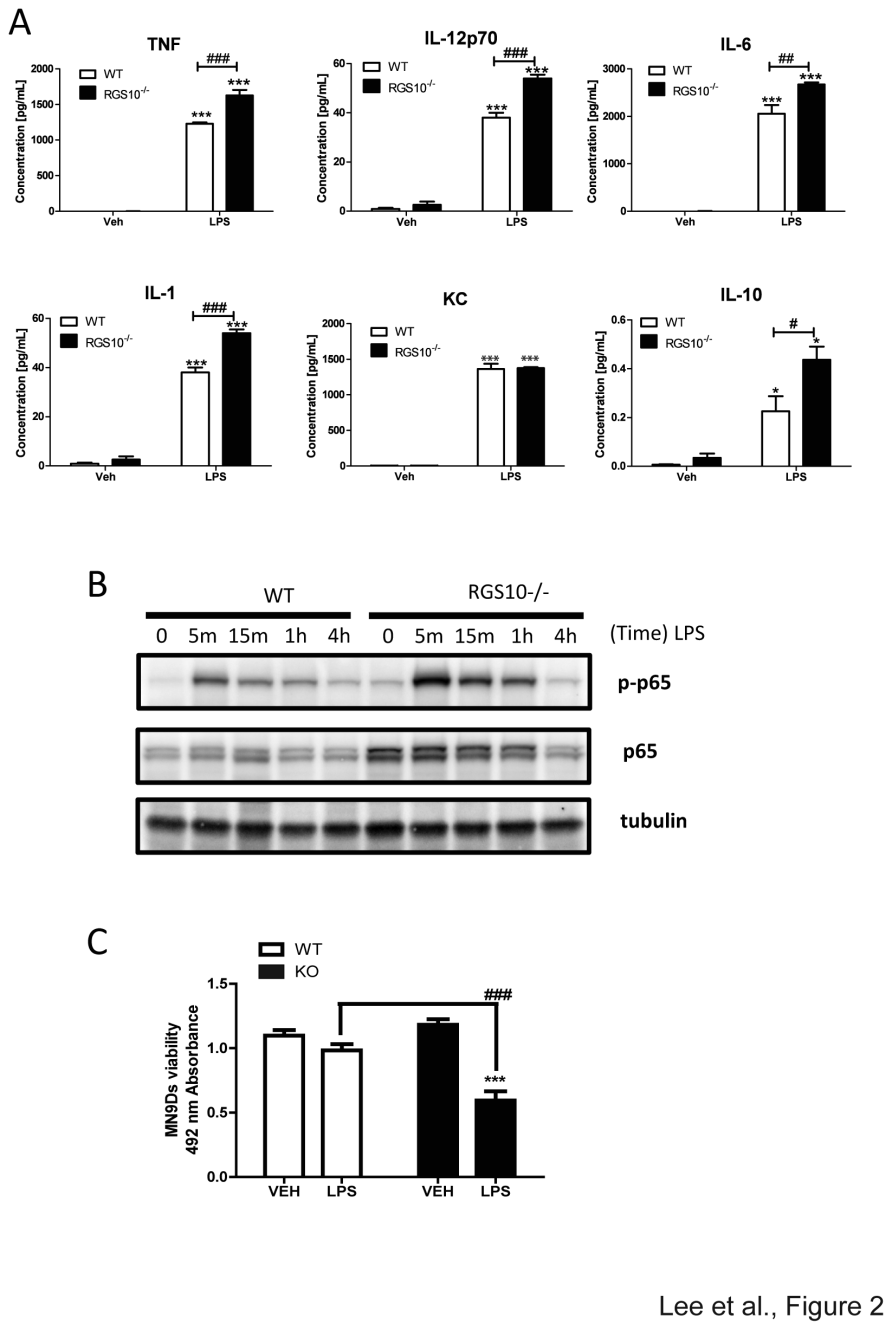
*Rgs10-/*- macrophages are hyper-responsive to LPS. A) Levels of pro-inflammatory cytokines and chemokines (TNF, IL-12 p40, IL-6, IL-1b, KC, IL-10) produced by wild type and *Rgs10-/*- BMDMs. Data are from one experiment representative of three independent experiments. Cells were plated and treated with LPS (100 ng/mL) for 24 hours as indicated in Materials and Methods. Conditioned medium was analyzed by Multiplexed immunoassay ELISA system (Meso Scale Discovery). Statistical analysis is described in Materials and Methods. *, *p* < 0.05;***p <0.001 compared within the group. ***^#^***, *p* < 0.05; ^##^, *p*< 0.01; ^###^ *p* <0.001 compared between the groups. B) Western blot analysis data of phospho-p65RelA and p65RelA expression in *Rgs10-/*- BMDMs after LPS (100 ng/mL) stimulation as indicated time. Data are from one experiment representative of three independent experiments. C) Conditioned media from BMDMs treated with LPS (100 ng/mL) for 24 hours were collected. MN9D neuroblastoma cells were treated with BMDM conditioned media for 48 hours. Cytotoxicity was measured by the MTS assay as described in Materials and Methods section. Values shown represent group means (n=4) ± S.E.M from one experiment representative of two independent experiments. *** denotes significant differences between vehicle and LPS within the group at p < 0.001 respectively. ***^##^*** denotes significant differences between groups at ^###^p < 0.001.

M1 macrophages produce pro-inflammatory cytokines and nitric oxide (NO) that are critical for immune responses [16]. Since RGS10 expression was attenuated by an M1 stimulus (LPS), we posited that this reduction in expression may be important for the development of the M1 phenotype. To investigate the role of RGS10 in M1 activation, we used *in vitro* generated BMDMs from WT and *Rgs10-/-* mice. We treated macrophages with LPS and measured the levels of cytokines and chemokines secreted into the conditioned media. We found that LPS induced secretion of most of the cytokines measured in our assay to a greater extent (TNF, IL-1, IL-6, IL-12p70, and IL-10) in *Rgs10-/-* macrophages relative to WT macrophages but not KC (Figure 2A). Because the NF-κB pathway is critical for LPS-induced pro-inflammatory cytokine production, we assessed whether this pathway was significantly affected by the loss of RGS10. LPS stimulation of *Rgs10-/- m*acrophages resulted in a modest increase in p65RelA phosphorylation relative to WT as well as a detectable increase in total p65RelA expression (Figure 2B). To determine whether increased levels of pro-inflammatory cytokines were sufficient to augment cytotoxicity, we performed target-effector assays as described under Materials and Methods. We found that conditioned media from LPS-treated *Rgs10-/-* macrophages exerted higher cytotoxicity on MN9D dopaminergic neuroblastoma cells (Figure 2C). However, we could not confirm the direct interaction of RGS10 protein with components of NF-κB signaling (p65 RelA or p50) using biochemical approaches (data not shown); yet our findings support a role for RGS10 in regulation of p65RelA expression. Taken together, our data suggest that RGS10 negatively regulates M1 macrophage activation by limiting NF-κB signaling pathway activation and the resulting cytokine production is responsible for enhanced cytotoxicity on neuronal cells. 

### *Rgs10-/-* macrophages display attenuated mRNA expression of M2 activation markers in response to IL-4

**Figure 3:**
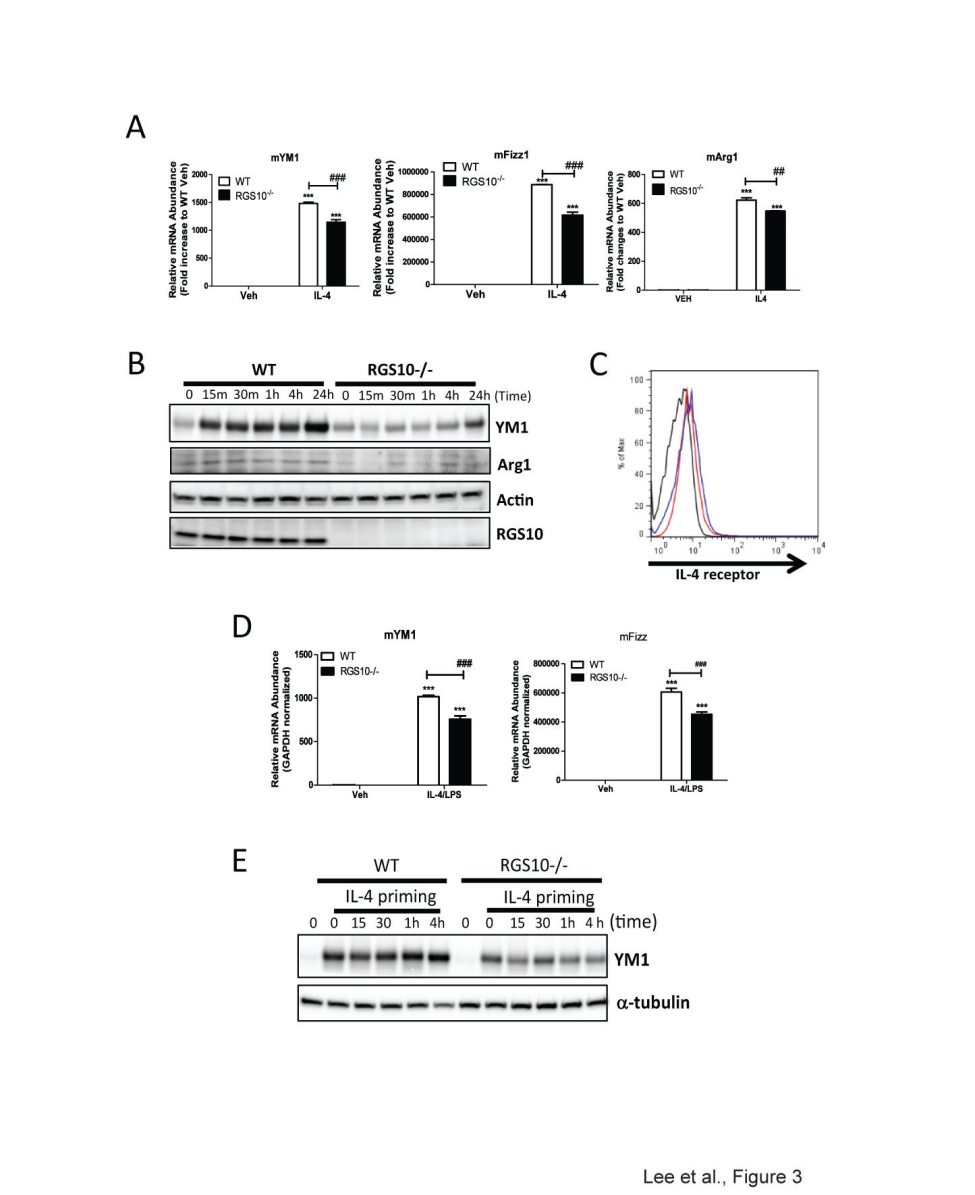
*Rgs10-/*- bone marrow derived macrophages (BMDMs) exhibit attenuated M2 activation phenotype. A) mRNA expression of YM1, Fizz1 and Arg1 mRNA from BMDMs treated with IL-4 (10 ng/mL) for 4 hours. Data are from one experiment representative of three independent experiments. B) Western blot analysis of YM1, Arg 1 and RGS10 proten expression in WT and *Rgs10-/*- BMDMs after IL-4 (10 ng/mL) treatment for the indicated times. C) IL-4R expression (with isotype control antibody in black) in CD45+CD11b+CD14+ BMDMs from WT (blue) or *Rgs10-/*- (red) mice. D) mRNA levels of YM1 and FIZZ1 in BMDMs upon LPS stimulation after IL-4 (10 ng/mL) priming for 48hours. Statistical analysis is described in Materials and Methods. **, *p* < 0.01;***p <0.001 compared within the group. ***^##^***, *p*< 0.01; ^###^ *p* <0.001 compared between the groups. E) BMDMs were treated with LPS for the indicated times after IL-4 (10 ng/mL) priming for 48hours. Western blot analysis of YM1 protein in BMDMs from WT or *Rgs10-/*- mice. Data are from one experiment representative of three independent experiments.

A key mechanism by which the effects of IL-4 or IL-13 are mediated is activation of the STAT6 pathway [17]. IL-4 induces expression of target genes characteristic of the M2 phenotype, including arginase-1 (*Arg1*), *Fizz 1*, *Ym1*, and other molecules that mediate several critical functions. To assess alternative activation in our cells, we primed BMDMs with IL-4 treatment. Markers of alternative activation were analyzed by real-time RT-PCR and western blot analysis. As shown in Figure 3, expression of YM1, Arg1 and Fizz1 mRNA was significantly increased in response to IL-4 in WT BMDMs. However, *Rgs10-/-* macrophages displayed significantly reduced expression of M2 markers (Figure 3A), indicating that RGS10 is required for optimal M2 activation. Concordant effects were seen at the protein level (Figure 3B). To rule out the possibility that the attenuated M2 response of *Rgs10-/-* macrophages could be due to reduced surface expression of IL-4 receptor, we performed flow cytometry analysis. BMDMs from WT and *Rgs10-/-* mice displayed similar IL-4 receptor expression at baseline (Figure 3C); therefore, we concluded that differences in surface expression of IL-4 receptor did not account for different M2 gene expression responses in *Rgs10-/-* vs. WT BMDMs. Our data indicate that RGS10 is required for optimal macrophage responses to M2 stimuli such as IL-4. Although macrophages treated with IL-4 are considered alternatively activated, IL-4 is considered a priming cytokine rather than an activating stimulus. Although both cytokines frequently induced opposing effects on gene transcription, the subsequent activation of bone marrow-derived macrophages by LPS produced a strong, priming-dependent pro-inflammatory response in both macrophage types. Therefore, we examined whether RGS10 loss affected the response to the subsequent TLR4-activation on IL-4-primed macrophages. Our data indicate that TLR4 activation on IL-4 primed *Rgs10-/-* macrophages relative to WT macrophages also results in attenuated expressions of alternative activation markers as measured by mRNA and protein levels (Figure 3D and E). 

**Figure 4:**
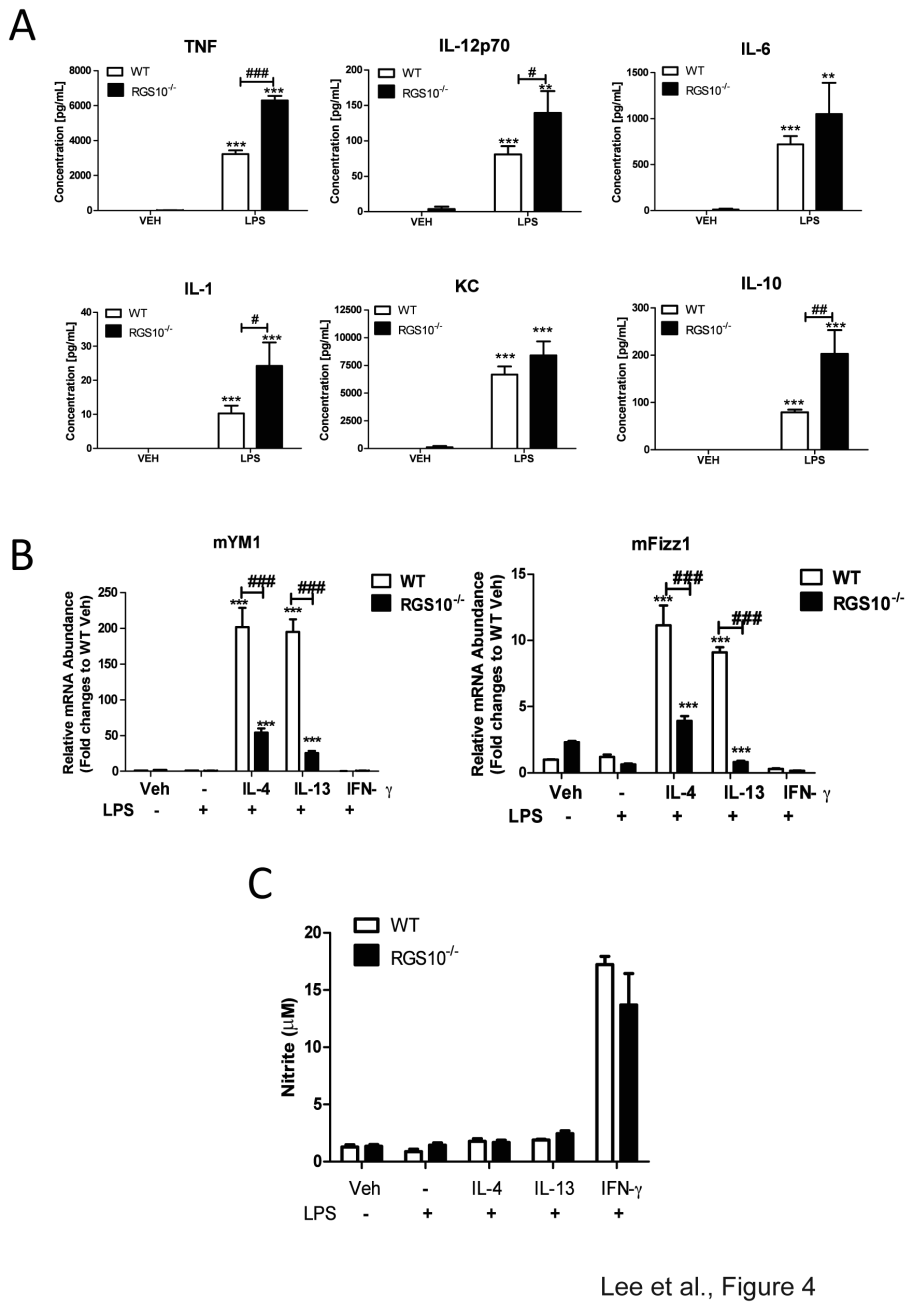
*Rgs10-/*- peritoneal macrophages display exaggerated classical activation and impaired alternative activation phenotypes. A) Production of cytokines and chemokines (TNF, IL-12 p40, IL-6, IL-1b, KC, IL-10) by WT and *Rgs10-/*- peritoneal macrophages. Peritoneal macrophages were isolated and treated with LPS (100 ng/mL) for 24 hours. Cultured medium was analyzed by Multiplexed immunoassay ELISA system (Meso Scale Discovery). Data are from one experiment representative of three independent experiments. B) Peritoneal macrophages were treated for 48 hours with IL-4, IL-13 or IFNγ (10 ng/mL) priming and challenged with LPS (100 ng/mL) for 24 hours. Levels of YM1, Fizz1 and Arg1 were determined by quantitative real-time PCR. C) Nitrite release was measured using the Griess reagent assay. Statistical analysis is described in Materials and Methods. **, *p* < 0.01;***p <0.001 compared within the group. ***^##^***, *p*< 0.01; ^###^ *p* <0.001 compared between the groups.

To confirm and extend our observations, we performed similar experiments on freshly isolated *in-vivo* differentiated peritoneal macrophages from WT and *Rgs10-/-* mice. In agreement with results in BMDMs, *Rgs10-/-* peritoneal macrophages produced higher levels of pro-inflammatory cytokines including TNF, IL-1β, IL-6, IL-12p70, and IL-10 relative to WT macrophages but not KC (Figure 4A) in response to LPS treatment. We also found that *Rgs10-/-* peritoneal macrophages displayed attenuated M2 responses relative to WT cells. Levels of YM1 and Fizz1 mRNA expression were significantly lower in *Rgs10-/-* peritoneal macrophages compared to WT (Figure 4B). Lastly, we also measured nitric oxide (NO) production as a part of the M1 response. Interestingly, *Rgs10-/-* macrophages did not exhibit enhanced NO production following IFN-γ and LPS stimulation as assessed by Griess reaction (Figure 4C). Together, these data indicate that an important function of RGS10 in macrophages is to limit pro-inflammatory cytokine production but not NO generation during M1 activation responses. 

### Phagocytosis and Chemotaxis in *Rgs10-/-* macrophages

**Figure 5:**
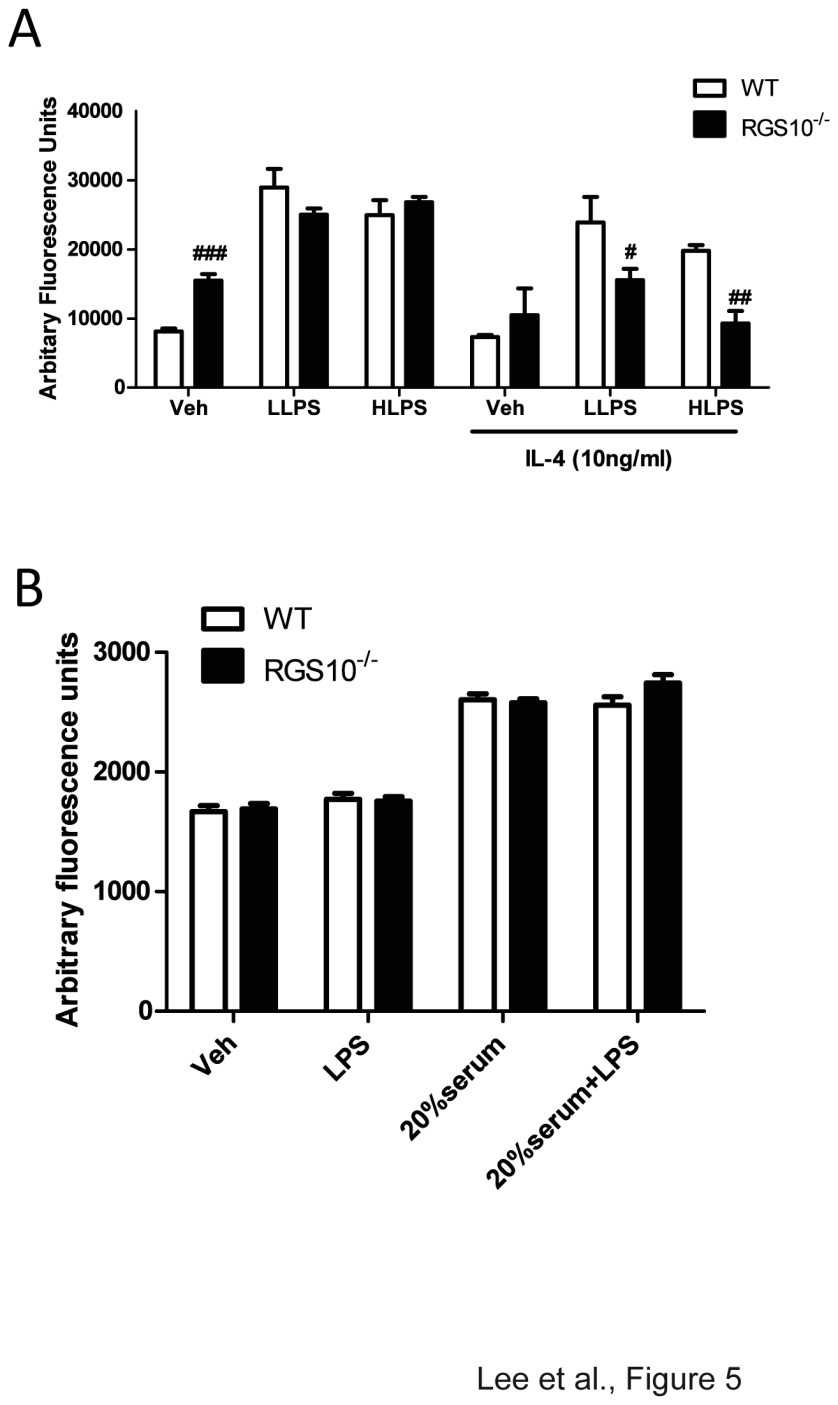
*Rgs10-/*- macrophages exhibit blunted phagocytic activity but normal chemotaxis. A) Phagocytic activity upon exposure to *Escherichia coli* (*E. coli*) particles is reduced in *Rgs10-/*- macrophages compared to WT macrophages. BMDMs were primed with IL-4 (10 ng/mL) for 48 hours. Each experimental condition was performed in quadruplicate. B) Chemotaxis of *Rgs10-/*- macrophages induced by LPS (1 µg/mL) or FBS (20%) is indistinguishable from that of WT macrophages. Data are from one experiment representative of three independent experiments. Cells were treated as described in Materials and Methods. Statistical analysis is described in Materials and Methods. **^*#*^**, p<0.05; ^##^, *p*< 0.01; ^###^ *p* <0.001 compared between the groups.

Based on results that indicated enhanced M1 polarization phenotype and attenuated M2 polarization in *Rgs10-/-* macrophages, we next examined the extent to which other whether macrophage effector functions were impaired in *Rgs10-/-* macrophages relative to WT cells. Phagocytic activity was measured using the Vibrant Phagocytosis Assay using fluorescently labeled *E. coli* particles. Interestingly, *Rgs10-/-* macrophages exhibited greater phagocytic activity at baseline but exhibited levels of phagocytosis similar to those in WT cells in response to LPS; however, IL-4 priming revealed blunted phagocytosis by *Rgs10-/-* macrophages in response to LPS (Figure 5A). Since RGS10 presumably functions as a GAP, it could regulate chemotaxis through direct coupling to chemokine receptors which are G-protein coupled receptors. We measured the chemotactic response of WT versus *Rgs10-/-* macrophages to serum-containing medium or serum plus LPS. No difference in chemotactic responses due to genotype was found (Figure 5B). Taken together, these findings support the conclusion that RGS10 is an important regulator of M1/M2 balance and primarily functions in an anti-inflammatory fashion in activated macrophages by limiting production of pro-inflammatory cytokines during periods of inflammatory stress. 

## Discussion

Previous work from our group demonstrated that LPS-treated *Rgs10-/-* brain microglia produce significantly higher amounts of pro-inflammatory cytokines including TNF, IL-1β, IL-6, IL-10, IL-12, and the chemokine CXCL1 compared to WT brain microglia. Moreover, increased pro-inflammatory cytokines in conditioned medium induced the robust death of dopaminergic neuroblastoma cells (MN9Ds) [13]. RGS10 is also expressed in immune organs including the lymph nodes and thymus; yet its function in immune cells remains poorly defined. Although microglia are brain-resident myeloid cells and thus share many similarities in function and express many surface markers in common with peripheral macrophages, considerable differences between these cell types have been reported [18]. Specifically, an elegant fate mapping study revealed that microglia originate from yolk-sac myeloid progenitors as opposed to the bone-marrow progenitors that give rise to monocytes and eventually peripheral macrophages [19]. Given these important differences, we investigated whether RGS10 also played a critical role in in macrophage activation responses. We found that *Rgs10-/-* macrophages displayed not only hypersensitivity to bacterial products (i.e. LPS) by increasing expression of genes typically associated with classical activation, but also displayed a reduced IL-4-induced alternative activation gene expression profile. It is well known that IL-4 initiates a cytoplasmic signaling cascade that culminates in tyrosine phosphorylation of transcription factor STAT6 [20]. In turn, phosphorylation of STAT6 induces expression of its target genes, including the alternative activation markers YM1 and Arg1, and regulatory factors such as the peroxisome proliferator-activated receptors (PPARs) and PPAR co-activator 1β (PGC-1β) [21,22]. Importantly, we confirmed using flow cytometry that the reduced IL-4 responsiveness of *Rgs10-/-* macrophages was not due to a decrease in expression of IL-4 receptors. Recently, studies from several laboratories support a critical role for nuclear receptors, PPARs, and PGC-1β in regulating M2 activation [23–25]. Specifically, it has been shown that PPARδ is required for professional phagocytosis in macrophages [26]. Functionally, activation of PPARδ induced the expression of regulator of G protein signaling (RGS) genes 1, 3, 4, 5, 10, 16, and 18 as PPARδ-regulated targets in the macrophage, suggesting modulation of G protein-coupled pathways as a common regulatory mechanism of this receptor [27]. Interestingly, our gene array data also revealed that baseline mRNA expression of PPARδ was 11-fold lower in *Rgs10-/-* microglia compared to WT microglia (data not shown), suggesting that there may be reciprocal regulation of PPARδ and RGS family protein expression that may contribute to the blunted phagocytic activity of *Rgs10-/-* macrophages. 

RGS10 was originally identified as a GTPase Activating Protein (GAP) that regulates heterotrimeric G protein signaling at the plasma membrane [9,32]. However, our findings as well as those of other groups have shown that RGS10 protein translocates to the nucleus and is found in high abundance at other intracellular sites [11,12], raising the interesting possibility that RGS10 possess functions that go beyond regulating heterotrimeric G-protein signaling at the plasma membrane. Our data is consistent with this idea and suggest that RGS10 might function to regulate macrophage activation responses through mechanisms that include changes in gene transcription associated with classical versus alternative activation. Given that RGS10 is the most soluble of the RGS proteins and its cellular distribution is both nuclear and cytoplasmic, it will be important to establish whether RGS10 might function to regulate macrophage stress responses through non-GAP mechanisms, such as changes in gene transcription and/or in cellular calcium regulation in addition to its GAP activity at the plasma membrane. Ongoing biochemical studies of *Rgs10-/-* macrophages will extend these findings and elucidate the exact molecular and signaling mechanisms by which RGS10 modulates macrophage activation.
